# Cortical Signatures of Dyslexia and Remediation: An Intrinsic Functional Connectivity Approach

**DOI:** 10.1371/journal.pone.0055454

**Published:** 2013-02-11

**Authors:** Maki S. Koyama, Adriana Di Martino, Clare Kelly, Devika R. Jutagir, Jessica Sunshine, Susan J. Schwartz, Francisco X. Castellanos, Michael P. Milham

**Affiliations:** 1 Rutgers University Center for Molecular and Behavioral Neuroscience, Newark, New Jersey, United States of America; 2 Phyllis Green and Randolph Cowen Institute for Pediatric Neuroscience, New York University Child Study Center, NYU Langone Medical Center, New York, New York, United States of America; 3 Center for the Developing Brain, Child Mind Institute, New York, New York, United States of America; 4 Nathan Kline Institute for Psychiatric Research, Orangeburg, New York, United States of America; University of Massachusetts Medical School, United States of America

## Abstract

This observational, cross-sectional study investigates cortical signatures of developmental dyslexia, particularly from the perspective of behavioral remediation. We employed resting-state fMRI, and compared intrinsic functional connectivity (iFC) patterns of known reading regions (seeds) among three dyslexia groups characterized by (a) no remediation (current reading and spelling deficits), (b) partial remediation (only reading deficit remediated), and (c) full remediation (both reading and spelling deficits remediated), and a group of age- and IQ-matched typically developing children (TDC) (total N = 44, age range = 7–15 years). We observed significant group differences in iFC of two seeds located in the left posterior reading network – left intraparietal sulcus (L.IPS) and left fusiform gyrus (L.FFG). Specifically, iFC between L.IPS and left middle frontal gyrus was significantly weaker in all dyslexia groups, irrespective of remediation status/literacy competence, suggesting that persistent dysfunction in the fronto-parietal attention network characterizes dyslexia. Additionally, relative to both TDC and the no remediation group, the remediation groups exhibited stronger iFC between L.FFG and right middle occipital gyrus (R.MOG). The full remediation group also exhibited stronger negative iFC between the same L.FFG seed and right medial prefrontal cortex (R.MPFC), a core region of the default network These results suggest that behavioral remediation may be associated with compensatory changes anchored in L.FFG, which reflect atypically stronger coupling between posterior visual regions (L.FFG-R.MOG) and greater functional segregation between task-positive and task-negative regions (L.FFG-R.MPFC). These findings were bolstered by significant relationships between the strength of the identified functional connections and literacy scores. We conclude that examining iFC can reveal cortical signatures of dyslexia with particular promise for monitoring neural changes associated with behavioral remediation.

## Introduction

Although the phonological deficit hypothesis (e.g., [1]–[2]) is the dominant explanatory theory of developmental dyslexia (a reading disorder), impaired phonological processing alone cannot explain the entirety of clinical symptomatology in the disorder. The majority of individuals with dyslexia suffer from multiple deficits in addition to or independent of phonological deficits [3]–[7]. Beyond abnormalities in phonological processing systems, emerging models of dyslexia highlight the potential contributions of dysfunction within systems supporting visual perceptual processing [8], auditory perceptual processing [9]–[11], rapid naming [12], and attention control [13]–[15]. In recent years, speculation has increased regarding cerebellar dysfunction in dyslexia [16]–[17]. Yet, with few exceptions [18]–[20], imaging studies of dyslexia have primarily focused on phonological deficits.

To help individuals with dyslexia improve literacy performance, substantial remediation efforts have been made, such as those targeting phonological deficits. These efforts have generally resulted in successful behavioral remediation (For review, see [21]). Recent task-based fMRI studies, using mainly phonological tasks, provide evidence of cortical activation changes associated with such behavioral changes (i.e., differences between before and after intervention) in individuals with dyslexia. Broadly, two cortical patterns appear to characterize behavioral remediation: 1) normalized (e.g., increased) activity detected in the known left hemisphere reading network (e.g., left temporoparietal and frontal regions) and 2) compensatory activity detected outside known reading networks (e.g., in right frontal regions) [22]–[26].

The goals of the present work are twofold. First, we aimed to conduct a broader examination of the cortical signatures of dyslexia by employing task-independent “resting-state” fMRI (R-fMRI). While task-based fMRI is of undoubted value, R-fMRI enables bypassing some of the limitations inherent to task-based developmental fMRI studies on reading and dyslexia (e.g., difficulties in developing reading tasks that can be equivocally performed by children in different age/grade groups). Second, we sought to obtain preliminary insights into the cortical signatures of behavioral remediation for reading and spelling deficits in individuals with dyslexia, using an observational, cross-sectional design. Specifically, we recruited individuals with documented evidence of a previous diagnosis of dyslexia, irrespective of the presence of prior remediation efforts or the current level of literacy competence. Consequently, participants varied substantially in literacy profiles, with some continuing to exhibit deficient reading and spelling, whereas others had apparently remediated reading difficulties and, in some cases, spelling deficiencies as well. To investigate the cortical signatures of this well-documented heterogeneity in dyslexia outcomes [27]–[28], we subdivided participants with a previous diagnosis of dyslexia into three groups: (a) children with current literacy deficits (no remediation), (b) children who remediated their reading deficits (partial remediation), and (c) children who remediated both reading and spelling deficits (full remediation).

Primary analyses focused on the examination of intrinsic functional connectivity (iFC) [29] in the aforementioned three dyslexia groups (i.e., no remediation, partial remediation, full remediation) and a matched typically developing comparison (TDC) group. Intrinsic functional connectivity is detected by examining inter-regional correlations in spontaneous low-frequency (<0.1 Hz) fluctuations in the R-fMRI signal [30]. This approach has delineated iFC patterns representing distinct functional networks [31]–[36] including reading networks [37]–[39]. Resting-state fMRI is increasingly being applied to studies of developmental disorders, including autism (e.g., [40]–[43]), attention deficit hyperactivity disorder (e.g., [44]–[47]), and Tourette syndrome [48]. In dyslexia, only one R-fMRI study has been conducted, focusing on the inter-hemispheric functional connectivity of inferior frontal regions [49].

**Figure 1:**
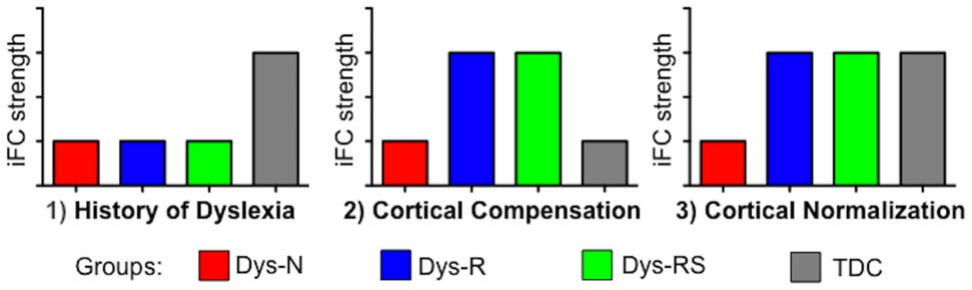
Hypothetical profiles of intrinsic functional connectivity. iFC = intrinsic Functional Connectivity, Dys-N = Dyslexia with No Remediation, Dys-R = Dyslexia with Reading Remediation, Dys-RS = Dyslexia with Reading and Spelling Remediation, TDC = Typically Developing Children.

In our previous work, we employed seed-based correlation analyses to assess iFC of regions commonly implicated in word reading [50]–[51] and demonstrated the utility of this approach for mapping reading networks in children and adults [38]–[39]. These reading circuits encompass regions previously identified as being dysfunctional in dyslexia (For review, see [52]). Here, we aimed to detect differences in iFC patterns of known reading regions as a function of the presence or absence of history of dyslexia (i.e., dyslexia vs. typically developing), as well as remediation status (i.e., no remediation, partial remediation, full remediation, typically developing). Accordingly, we predicted that we would detect three types of outcomes: 1) atypical iFC associated with a history of dyslexia (e.g., iFC common to all dyslexia groups and distinct from that of the TDC group), 2) altered iFC reflecting cortical compensation associated with behavioral remediation (e.g., iFC distinguishing the remediation groups from both the TDC group and the no remediation group), and 3) altered iFC reflecting cortical normalization associated with behavioral remediation (e.g., iFC distinguishing the remediation groups only from the no remediation group but not from the TDC group). These hypothetical iFC profiles are illustrated in Figure 1.

## Materials and Methods

### Participant Characteristics

Participants were native English-speaking children with (n = 33) and without (n = 11) a history of dyslexia (i.e., documented prior diagnosis of dyslexia). We recruited children with a history of dyslexia through referrals from the clinical services at the New York University Child Study Center and the New York International Dyslexia Association. Inclusion in dyslexia groups was based upon parental report of prior diagnosis of reading disorder in accord with DSM-IV or ICD-10, accompanied by prior written documentation. We enrolled a total of 33 children with a history of dyslexia. Among them, 22 exhibited partial (n = 11) or full (n = 11) literacy remediation based on standardized subtests of the Wechsler Individual Achievement Test – Second Edition (WIAT) [53]. Specifically, children with a history of dyslexia were classified as remediated if their current standard scores were above 85, corresponding to one standard deviation below the norm, on the WIAT Word Reading subscale (partial remediation), or on both the WIAT Word Reading and the WIAT Spelling subscales (full remediation). The remaining 11 individuals with current standard scores lower than 85 on both the WIAT Word Reading and the WIAT Spelling subscales constituted the no remediation group. Accordingly, there were 3 dyslexia groups: (1) children with current deficits in both reading and spelling (“Dys-N”: Dyslexia with no remediation), (2) children with a previous diagnosis of dyslexia but exhibiting no current reading deficit (“Dys-R”: Dyslexia with reading remediation), and (3) children with a previous diagnosis of dyslexia but exhibiting no current deficits in either reading or spelling (“Dys-RS”: Dyslexia with reading and spelling remediation).

According to parental report (and supporting documentation for some cases), children in the remediation groups had a history of one or more targeted interventions prior to the current study (e.g., the Orton Gillingham approach; http://www.ortonacademy.org, Wilson Language Training; http://www.wilsonlanguage.com, or miscellaneous school intervention efforts). In contrast, none of the children with current literacy deficits (no remediation group) had a history of targeted dyslexia intervention training. This pattern suggests that behavioral remediation is related, at least in part, to targeted intervention training, although the specific forms of intervention varied and are not a focus of examination here due to limited sample size. Importantly, prior written documentation served to verify literacy impairment in the two remediation groups (standard scores lower than 85 on any type of standardized literacy test prior to remediation). Similarly, the written documentation provided evidence that the majority of these children had exhibited deficits in phonological skills (e.g., phonological awareness), consistent with the phonological deficit hypothesis (e.g., [54]).

For a control group, eleven typically developing children (TDC) were selected from a larger pool of children participating in ongoing studies at NYU Child Study Center. In the TDC group, all children’s standard scores were above 85 on both WIAT Word Reading and WIAT Spelling, confirming lack of impairments in either reading or spelling. They had no previous or current DSM-IV-TR diagnoses of psychiatric disorders based on the Schedule for Affective Disorders and Schizophrenia for School-Age Children–Present and Lifetime Version [KSADS-PL: 55], which was administered to parents and child participants separately. The KSADS-PL was not administered to children with a history of dyslexia and/or their parents. Nevertheless, we ascertained whether these children had received a previous diagnosis of disorders other than dyslexia, such as attention deficit hyperactivity disorder (ADHD), through informal interviews with parents of dyslexic children and by reviewing documentation of prior clinical evaluations whenever available (Three out of 33 children with a history of dyslexia had been previously diagnosed with ADHD).

**Figure 2:**
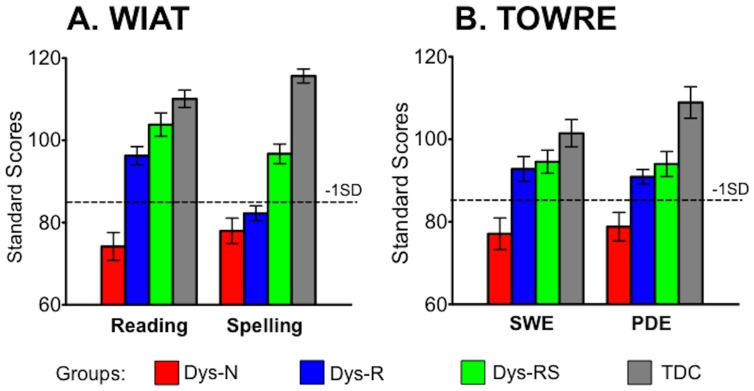
Group mean and standard deviation of literacy performance. (A) WIAT = Wechsler Individual Achievement Test and (B) TOWRE = Test of Word Reading Efficiency, n = 11 for each group. SWE = Sight Word Reading Efficiency, PDE = Phonemic Decoding Efficiency, SD = Standard Deviation, Dys-N = Dyslexia with No Remediation, Dys-R = Dyslexia with Reading Remediation, Dys-RS = Dyslexia with Reading and Spelling Remediation, TDC = Typically Developing Children: -1SD represents a standard score of 85, one standard deviation below the norm.

Children in each of the 4 groups were group-matched on age (overall mean age = 12.2±2.3 years: range = 7.7–15.7 years), sex, estimated full-scale IQ, and handedness. The Wechsler Abbreviated Scale of Intelligence (WASI) [56] provided full-scale IQ estimates, and all participants had full-scale IQ estimates above 85. We also administered two subtests of the Test of Word Reading Efficiency (TOWRE) [57]) – Sight Word Efficiency (SWE) and Phonemic Decoding Efficiency (PDE). Further literacy-related measures were administered to children in the dyslexia groups, including two subtests of the Comprehensive Test of Phonological Processing (CTOPP) [58] – Elision (a phonological awareness test) and Nonword Repetition (a phonological short-term memory test), as well as WIAT Reading Comprehension. Additionally, given the increased incidence of ADHD symptomatology in individuals with dyslexia (e.g., [59]–[60]), we asked all parents to complete the Conners Parent Rating Scales–Revised Long Version (CPRS-R-L: [61]) which addresses ADHD symptoms. Table 1 provides demographic and cognitive measures for each group, and Figure 2 illustrates their literacy profiles.

**Table 1:** Demographic and cognitive profiles (means and standard deviation) for each group.

	Dyslexia-N	Dyslexia-R	Dyslexia-RS	TDC	F or *X* ^2^
Age (years)	12.4 (2.1)	11.8 (2.2)	12.6 (2.9)	12.2 (2.3)	N.S.
Gender (n)	6M/5F	5M/6F	5M/6F	6M/5F	N.S.
Handedness	0L/11R	0L/11R	0L/11R	0L/11R	N.S.
WASI Full IQ(ss)	108.1 (6.9)	101.3 (9.8)	108.1 (5.1)	106.5(5.8)	N.S.
WIAT Read(ss)	74.2 (11.3)	96.3 (7.3)	103.8 (9.3)	110.1 (7.0)	F = 34.1***
WIAT Spell(ss)	78.0 (10.2)	82.3 (5.9)	96.7 (7.9)	115.6(5.76)	F = 61.3***
WIAT RC (ss)	94.6 (9.9)	97.4 (8.3)	113.5(14.2)	N.A.	F = 9.4***
TOWRE SWE(ss)	77.1 (12.7)	92.8 (9.9)	94.6 (9.2)	101.5 (11.0)	F = 10.0***
TOWRE PDE(ss)	78.8 (11.4)	90.9 (5.9)	94.0 (10.0)	109.0 (12.6)	F = 15.8***
CTOPP Elision(ss) ^†^	7.9 (2.7)	12.4 (1.9)	11.6 (2.8)	N.A.	F = 13.5***
CTOPP NWR(ss)^†^	6.9 (2.4)	7.7 (2.2)	9.4 (2.5)	N.A.	F = 3.6*
CPRS-IA (ts)	57.1 (11.3)	62.6 (15.2)	53.4 (9.4)	44.8 (5.1)	F = 5.3**
CPRS-HI (ts)	56.0 (6.5)	55.4 (13.4)	50.8 (9.9)	45.5 (4.1)	F = 3.1*

n = 11 for each group, *p<0.05, **p<0.01, ***p<0.001: N.A. = Not Available, N.S. = Not Significant; Dys-N = Dyslexia with No Remediation, Dys-R = Dyslexia with Reading Remediation, Dys-RS = Dyslexia with Reading and Spelling Remediation, TDC = Typically Developing Children, ss = standard score (mean = 100, standard deviation = 15), ts = t score, M = Male, F = Female, L = Left, R = Right, WASI = *Wechsler Abbreviated Scale of Intelligence,* WIAT = Wechsler Individual Achievement Test – Second Edition, RC = Reading Comprehension, TOWRE = Test of Word Reading Efficiency, SWE = Sight Word Reading Efficiency, PDE = Phonemic Decoding Efficiency, CTOPP = Comprehensive Test of Phonological Processing, NWR = NonWord Repetition, CPRS-IA = *Conners’ Parent Rating Scales DSM-IV Symptom Inattentive index, CPRS-HI = Conners’ Parent Rating Scales DSM-IV Symptom Hyperactive-Impulsive index:* ^†^ Standard scores for CTOPP measures are based on a mean of 10 with standard deviation of 3.

To help anchor the results of the current study, we performed a secondary analysis that aimed to verify that iFC patterns derived from the TDC group represent normative/typical iFC profiles. We examined iFC in an age- and IQ-matched, independent group of TDC (n = 25, mean age = 11.7±1.6 years), as well as in a group of typical adults (TA; n = 25, mean age: 31.6±8.2), both of which were studied previously [39]. This enabled us to examine whether iFC strength for regions showing significant group effects was normative in the two additional typical groups. The current study was approved by the institutional review board of New York University (NYU) School of Medicine, and all parents and children provided written informed consent and assent.

### MRI Data Acquisition

MRI data were collected on a Siemens Allegra 3.0 T scanner at the NYU Center for Brain Imaging. We collected resting-state fMRI data in an oblique plane using a customized multi-echo planar imaging (EPI): 180 whole-brain volumes; TR = 2000 ms; effective TE = 33 ms; flip angle = 90°; 33 contiguous 4 mm slices, matrix = 80×64; acquisition voxel size = 3×3×4 mm. This sequence uses the dead time that precedes the readout in EPI sequences with normal TEs (∼33 ms) to collect additional images at several echo times. However, the information from these additional echo times in not used in our reconstruction of the data. For spatial normalization and localization, we also acquired a high-resolution T1-weighted magnetization prepared gradient echo sequence (TR = 2530 ms; TE = 3.25 ms; TI = 1100 ms; flip angle = 7°; 128 slices; field of view = 256 mm).

### Data Preprocessing

For each participant, image preprocessing was carried out using a combination of AFNI (http://afni.nimh.nih.gov) [62] and FSL (http://www.fmrib.ox.ac.uk/fsl/). Scripts containing data processing steps similar to those employed here have been released as part of the ‘1000 Functional Connectomes Project [63] (http://www.nitrc.org/projects/fcon_1000). Preprocessing comprised: 1) discarding the first 4 EPI volumes from each scan to allow for signal equilibration, 2) slice timing correction for interleaved acquisitions, 3) 3D motion correction with Fourier interpolation, 4) time series despiking (detection and reduction of extreme time series outliers), 5) spatial smoothing (6 mm FWHM Gaussian kernel), 6) mean-based intensity normalization of all volumes by the same factor, 7) temporal band-pass filtering (0.009–0.11 Hz), and 8) linear and quadratic detrending. Registration of each participant’s high-resolution anatomical image to standard 2×2×2 mm MNI space was accomplished in two steps [64]. First, a 12-degrees-of-freedom linear affine transformation was computed using FLIRT [65]–[66]. This transformation was then refined using FNIRT nonlinear registration [64]. Linear registration of each participant’s functional time series to the high-resolution structural image was performed using FLIRT. This functional-to-anatomical co-registration was improved by intermediate registration to a low-resolution image and b0 warping. To control for potential effects of physiological processes (e.g., fluctuations related to cardiac and respiratory cycles) [67]–[68], motion and large-scale neural signals present throughout the brain [69], each participant’s preprocessed data was regressed on nine nuisance covariates (signals from white matter, cerebrospinal fluid, the global signal, and six motion parameters). Each participant’s resultant 4D residual volume was spatially normalized by applying the previously computed transformation to 2 mm MNI space.

### Seed Regions-of-interest (ROIs)

**Figure 3:**
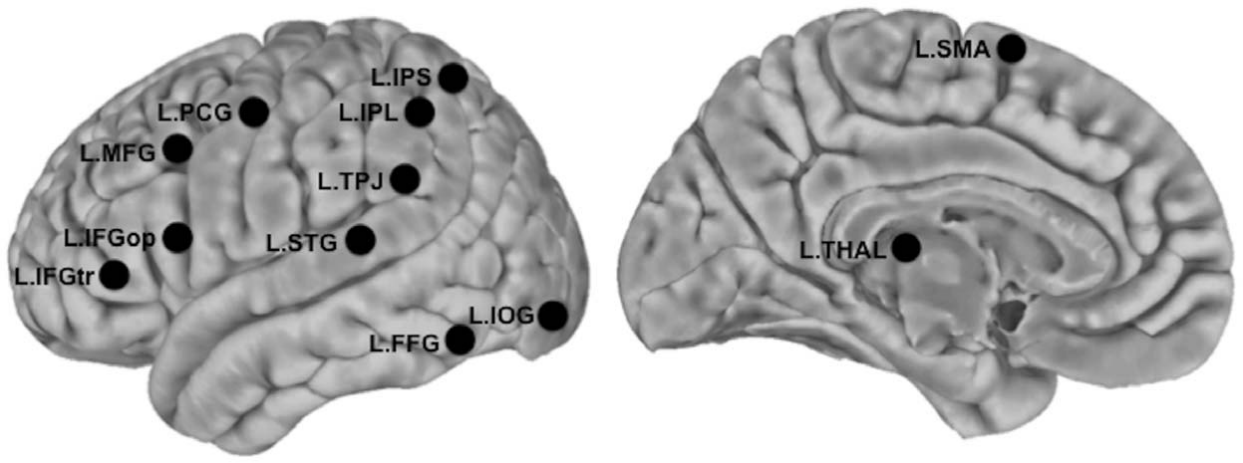
Locations of seed ROIs. Seeds are presented as overlaid black circles. MNI coordinates of these seeds are provided in Table 2. L.IOC = Left Inferior Occipital Gyrus, L.FFG = Left Fusiform Gyrus, L.STG = Left Superior Temporal Gyrus, L.TPJ = Left Temporoparietal Junction, L.IPS = Left Inferior Parietal Lobule, L.IPS = Left Intraparietal Sulcus, L.PCG = Left Precentral Gyrus, L.SMA = Left Supplementary Motor Area, L.IFGop = Left Inferior Frontal Gyrus pars Opercularis, L.IFGtr = Left Inferior Frontal Gyrus pars Triangularis, L.MFG = Left Middle Frontal Gyrus, L.THAL = Left Thalamus.

We examined functional connectivity associated with 12 regions of interest (ROIs), including 11 regions that had been derived from meta-analyses of reading in children [51] and adults [50] and validated in a previous R-fMRI study [39]. One additional region, the left inferior parietal lobule (L.IPL), was added based on the most recent meta-analysis of children with dyslexia [52]; the remainder of the regions in the meta-analysis were already included in our previous work [39]. The 12 ROIs in the current study were: 1) left inferior occipital gyrus (L.IOG), 2) left posterior fusiform gyrus (L.FFG), 3) left posterior superior temporal gyrus (L.STG), 4) left temporoparietal junction (L.TPJ), 6) left inferior parietal lobule (L.IPL), 7) left intraparietal sulcus (L.IPS), 8) left dorsal precentral gyrus (L.PCG), 9) left supplementary motor area (L.SMA), 8) left inferior frontal gyrus pars opercularis (L.IFGop), 10) left inferior frontal gyrus pars triangularis (L.IFGtr), 11) left middle frontal gyrus (L.MFG), and 12) left thalamus (L.THAL). Each ROI was a spherical seed (6 mm radius in 2 mm standard space) [39], centered on the MNI coordinates listed in Table 2. Figure 3 shows the spatial locations of the seeds.

**Table 2:** MNI coordinates of the regions of interest.

Regions-of-Interest			MNI (x, y, z)
***Occipital***			
	**IOG**	Inferior Occipital Gyrus (posterior)	**−25 −87 −10**
	**FFG**	Fusiform Gyrus (posterior)	**−48 −57 –20**
***Temporal***			
	**STG**	Superior temporal gyrus (posterior)	**−53 −31 9**
	**TPJ**	Temporoparietal Junction	**−59 −45 15**
***Parietal***			
	**IPL**	Inferior parietal lobule	**−40 −48 42**
	**IPS**	Intraparietal sulcus (posterior)	**−30 −58 48**
***Motor***			
	**PCG**	Precentral gyrus (dorsal)	**−48 −12 45**
	**SMA**	Supplementary motor area	**−4 10 58**
***Frontal***			
	**IFGop**	Inferior frontal gyrus (opercularis)	**−51 10 10**
	**IFGtr**	Inferior frontal gyrus (triangularis)	**−48 32 6**
	**MFG**	Middle frontal gyrus (ventral)	**−44 10 30**
***Subcortical***			
	**THAL**	Thalamus	**−10 −14 8**

The seeds were selected based on meta-analyses of reading in typical adults [50] and typically developing children [51], as well as dyslexic children [52].

### Participant-level iFC Analyses

For each participant, the representative time series for each seed ROI was extracted from their 4D residuals volume in standard space by averaging the time series across all voxels within the ROI. This step ensured, to as great an extent as possible, that the mean time series were extracted from the same regions across participants. Calculation of the correlation between each seed ROI time series and that of every other brain voxel was performed in native space to minimize computational and storage demands. This procedure is consistent with our prior publications [70]–[72]. The resultant participant-level correlation maps were Fisher-r-to-z transformed, then transformed into MNI152 2 mm standard space for group-level analyses.

### Group-level iFC Analyses

For each seed ROI, group-level analyses were carried out using a random-effects ordinary least squares model. Whole-brain correction for multiple comparisons was performed using Gaussian Random Field Theory (min Z >2.3; cluster significance: p<0.05, corrected). To test the effect of group on iFC, we performed a one-way ANOVA, treating group as a 4-level factor (Dyslexia-N, Dyslexia-R, Dyslexia-RS, and TDC). Subsequently, regions exhibiting significant main effects of group on iFC were interrogated by post hoc analysis using unpaired t-tests to determine significant pair-wise differences between groups.

To account for the effects of head motion [73]–[76], we calculated frame-by-frame changes in head position, referred to as framewise displacement (FD), for each participant, and included mean FD values as a covariate in the group-level model. As detailed elsewhere [73], FD is calculated from the derivatives of the rigid body realignment estimates produced by the motion correction algorithm during fMRI preprocessing. Statistically, mean FD values neither differed among the four groups, F (3, 40) = 1.06, p = 0.38, (Figure S1.A), nor correlated with age (R^2^ = 0.01, p = 0.49) (Figure S1.B).

In addition to the primary group analysis, which included both global signal regression (GSreg) at the individual level and mean FD regression (FDreg) at the group level (i.e., +GSreg+FDreg), we performed several additional confirmatory group analyses. These were carried out in response to recent debates regarding R-fMRI analytic methods, particularly concerning the effects of micro-movements (e.g., [73]) and global signal regression [77]–[78]. Four confirmatory group analyses were conducted: 1) with GSreg at the individual level but no mean FDreg at the group level (+GSreg-FDreg), 2) with GSreg and removal (“scrubbing”) of volumes with FD larger than 0.5 mm at the individual level, without FDreg at the group level (+GSreg+Scrub), 3) without GSreg at the individual level but with mean FDreg at the group level (-GSreg+FDreg), 4) without GSreg at the individual level or mean FDreg at the group level (-GSreg-FDreg).

### Brain-behavior Relationships

We explored relationships between the strength of iFC among regions showing group effects and literacy performance (i.e., accuracy on WIAT Word Reading and WIAT Spelling subtests) as well as ADHD symptom scores (two CPRS DSM-IV Symptoms Subscales: Inattention and Hyperactivity-Impulsivity). Multiple regression analyses were performed to investigate whether individual differences in literacy performance (and ADHD symptoms) were associated with individual differences in the strength of iFC associated with each seed in each group, across dyslexia groups, and/or across all groups.

### Secondary Analysis

n the current study, the sample size for each group was equal but relatively small (n = 11 for each group). In order to verify that iFC patterns derived from the TDC group are representative of normative/typical iFC profiles, we performed unpaired t-tests comparing the strength of iFC between regions exhibiting significant group effects between all pairs of the following three groups; 1) the current TDC group (n = 11), 2) an age- and IQ- matched, independent TDC group (n = 25), and 3) a typical adult (TA) group (n = 25). The latter two groups were described previously [39]. We expected that the two TDC groups would not differ from one another. Group differences between each of the two TDC groups and the TA group would only be expected if the networks exhibiting significant dyslexia-related differences were also developmentally sensitive.

## Results

### Dyslexia Sample Characterization

One-way ANOVA showed that the four groups (i.e., TDC, Dys-N, Dys-R, and Dys-RS) did not differ significantly with respect to age, sex, estimated full-scale IQ, or handedness, but differed significantly on the literacy measures (i.e., WIAT Reading, WIAT Spelling, TOWRE SWE, and TOWRE PDE), as expected (Table 1 and Figure 2). Post-hoc analysis using unpaired t-tests revealed significant differences on these measures between each pair of groups; as expected, the Dys-N group performed significantly worse on all these measures than the TDC group (Supplementary Material1).

On other literacy-related measures (i.e., WIAT Reading Comprehension, CTOPP Elision, and CTOPP Nonword Repetition) specifically administered to children with a history of dyslexia, the three dyslexia groups also differed significantly (Table 1). Significant post-hoc differences between each pair of groups were noted (Supplementary Material1). Of note, all dyslexic children’s performance (except for one child in the Dys-N group) fell within the normal range on WIAT Reading Comprehension. However, some dyslexic children, particularly those in the Dys-N group, showed impairments (i.e., standard scores <8) on the CTOPP phonological measures.

When reading and spelling errors were examined, some children with a history of dyslexia, even in the remediation groups, made errors such as substitution of visually similar words (e.g., ‘useless’ pronounced as ‘unless’), omission (e.g., ‘climb’ spelled as ‘clim’), transposition (e.g., ‘receive’ spelled as ‘recieve’), and sounding out silent letters (e.g., ‘b’ pronounced in ‘subtle’). These errors, typically made by individuals with dyslexia, are considered to reflect inadequate phonological and/or orthographic representations of words [79].

Among the 33 children with dyslexia, a previously established diagnosis of ADHD was reported for only three participants. However, as expected for individuals with dyslexia [80], elevated levels of inattention and hyperactivity-impulsivity, indexed as T scores higher than 60 on the Conners’ Inattentive and Hyperactive-Impulsive subscales, respectively, were noted in these children: One-way ANOVA showed a significant main effect of group for both inattention and hyperactivity-impulsivity scores (Table 1), and post-hoc analysis revealed that both ADHD symptom domains were significantly elevated in the dyslexia groups relative to the TDC group (Material S1 and Figure S2.A). Consistent with recent evidence emphasizing the importance of attention in reading (For review, see [81]), inattention scores were negatively correlated with reading scores across all groups (R^2^ = 0.24, p<0.001), and across dyslexia groups (R^2^ = 0.12, p<0.05), particularly in Dys-R (R^2^ = 0.65, p<0.01) (Figure S2.B.1). A significant correlation between hyperactivity-impulsivity and reading scores was also observed across all groups (R^2^ = 0.13, p<0.05) (Figure S2.B.2). Accordingly, in addition to our primary analyses, we examined the association between each of these two measures of ADHD symptoms and iFC within the reading network (details below).

### Group Effects

**Figure 4:**
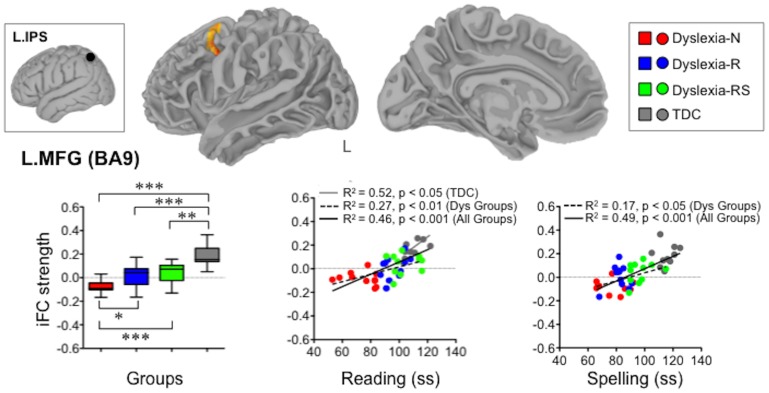
Atypical iFC between the left intraparietal sulcus (L.IPS) and left middle frontal gyrus (L.MFG) associated with historical diagnosis of dyslexia. The box-and-whisker plot depicts L.IPS-L.MFG iFC for each group and group differences, whereas the two scatterplots represent the relationships between the strength of this connection and reading as well as spelling scores. iFC = intrinsic Functional Connectivity, Dys-N = Dyslexia with No Remediation, Dys-R = Dyslexia with Reading Remediation, Dys-RS = Dyslexia with Reading and Spelling Remediation, TDC = Typically Developing Children, Dys = Dyslexia, ss = standard scores: ***p<0.001, **p<0.01, *p<0.05 (corrected): L.IPS-L.MFG iFC was weaker in all dyslexia groups relative to TDC.

**Figure 5:**
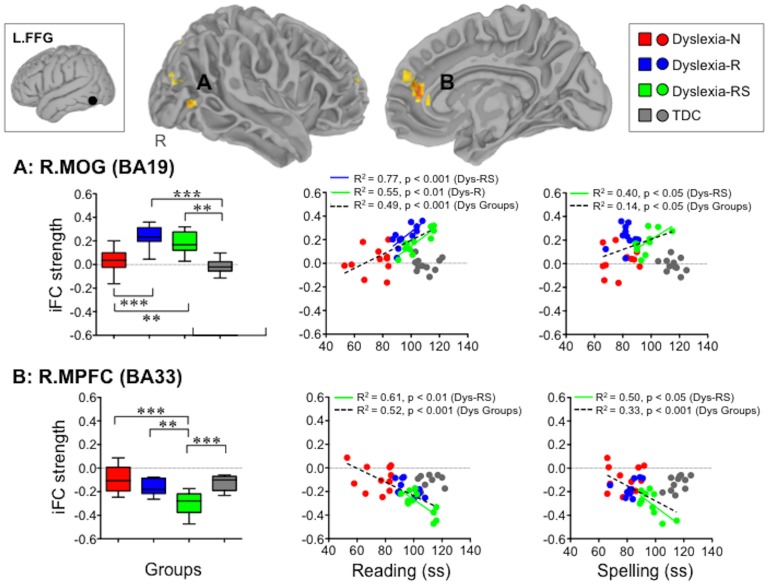
Compensatory changes in iFC of the left fusiform gyrus (L.FFG) in the remediation groups. The box-and-whisker plots depict (A) L.FFG-R.MOG iFC and (B) L.FFG-R.MPFC iFC for each group and group differences, whereas the scatterplots represent the relationships between the strength of these connections and reading as well as spelling scores. iFC = intrinsic Functional Connectivity, R.MOG = Right Middle Occipital Gyrus, R.MPFC = Right Medial Prefrontal Cortex, Dys-N = Dyslexia with No Remediation, Dys-R = Dyslexia with Reading Remediation, Dys-RS = Dyslexia with Reading and Spelling Remediation, TDC = Typically Developing Children, Dys = Dyslexia, ss = standard scores: ***p<0.001, **p<0.01 (corrected): Both the partial and full remediation groups exhibited stronger L.FFG-R.MOG iFC, relative to both DTC and Dys-N, whereas the full remediation group exhibited stronger negative L.FFG-R.MPFC iFC, relative to TDC, Dys-N, and Dys-R.

One-way ANOVA, with group as level (4 levels: no remediation, partial remediation, full remediation, and TDC), identified a significant main effect of group (Z >2.3; p<0.05, corrected) on iFC associated with two seeds in the posterior reading network – the left intraparietal sulcus (L.IPS) and left fusiform gyrus (L.FFG). Specifically, we observed a main effect of group on iFC between the L.IPS seed and left middle frontal gyrus (L.MFG, BA9; x = −34, y = 4, z = 40; see Figure 4), and on iFC between the L.FFG seed and the right middle occipital gyrus, extending towards the right intraparietal sulcus (R.MOG, BA19; x = 34, y = −70, z = 4; see Figure 5.A), as well as between the L.FFG seed and the right medial prefrontal cortex (R.MPFC, BA33; x = 14, y = 46, z = 10; see Figure 5.B), a region corresponding to the anterior region of the dorsal MPFC [82].

Of note, our findings were generally robust to the particular micro-movement correction strategy employed (i.e., regression of mean FD, “FDreg”; scrubbing at FD >0.5 mm, “Scrub”; see Figure S3). In contrast, dependencies on global signal regression (GSreg) were noted for iFC of the L.FFG seed but not that of the L.IPS seed: In the absence of GSreg, the L.FFG findings failed to survive multiple comparison correction, but were significant only at uncorrected p<0.05. Figure S4 illustrates such region-specific effects of GSreg on the group differences in these intrinsic functional connections. These findings suggest that GSreg served to account for unexplained variation in these connections, although resolution of this issue is beyond the scope of the current study. Accordingly, the remainder of the results section focuses on findings revealed by the analysis conducted with GSreg.

### Interrogation of Pairwise Group Differences

Statistical interrogation (i.e., pairwise comparisons via unpaired t-test) of regions exhibiting a main effect of group revealed two profiles – one associated with the historical diagnosis of dyslexia, regardless of remediation status (L.IPS-L.MFG), and one reflecting cortical compensation associated with behavioral remediation (L.FFG-R.MOG, L.FFG-R.MPFC).

### Atypical Connectivity Associated with Historical Diagnosis of Dyslexia

Post-hoc analysis showed that the iFC between the L.IPS seed and L.MFG was weaker in all dyslexia groups relative to TDC (Figure 4): Dys-N (t = 8.44, p<0.001), Dys-R (t = 4.42, p<0.001), and Dys-RS (t = 3.75, p<0.01). Among dyslexia groups, iFC between L.IPS and L.MFG was significantly weaker in Dys-N relative to both Dys-R (t = 2.69, p<0.05) and Dys-RS (t = 4.06, p<0.001). That is, amongst dyslexia groups, iFC between L.IPS and L.MFG was weakest in the no remediation group. Notably, all children in TDC group exhibited positive L.IPS-L.MFG connectivity, as contrasted with negative connectivity exhibited by the majority of children in the no remediation group.

Examination of dimensional relationships between iFC and literacy competence (i.e., reading and spelling) further highlighted the contributions of L.IPS-L.MFG functional connectivity to literacy. Specifically, multiple regression analyses revealed that iFC between L.IPS and L.MFG was positively correlated with literacy performance across all groups (Reading, R^2^ = 0.46, n = 44, p<0.001; Spelling, R^2^ = 0.49, n = 44, p<0.001; scatter plots in Figure 4). That is, children who exhibited stronger positive iFC between L.IPS and L.MFG tended to have higher scores in literacy performance. Such brain-behavior relationships were also noted across all dyslexia groups (Reading, R^2^ = 0.27, n = 33, p<0.01; Spelling, R^2^ = 0.17, n = 33, p<0.05). When examining individual groups, only the relationship between L.IPS-L.MFG iFC and reading performance within the TDC group reached statistical significance (R^2^ = 0.52, n = 11, p<0.05).

### Cortical Changes Associated with Behavioral Remediation

Post-hoc analysis revealed that the iFC patterns associated with the L.FFG seed differed significantly between the remediation groups and the remaining groups (i.e., the TDC group and the no remediation group) (Figure 5). First, the two remediation groups (the partial and full remediation groups) exhibited significantly stronger positive iFC between L.FFG and R.MOG, relative to TDC (Dys-R, t = 6.15, p<0.001; Dys-RS, t = 4.84, p<0.01), and relative to Dys-N (Dys-R, t = 5.30, p<0.001; Dys-RS, t = 3.39, p<0.01). No group difference was observed either between Dys-R and Dys-RS (t = 1.31) or between TDC and Dys-N (t = 0.85) (Figure 5.A). Second, Dys-RS (the full remediation group) exhibited stronger negative iFC between L.FFG and R.MPFC, relative to TDC (t = 4.61, p<0.001), Dys-N (t = 5.73, p<0.001), and Dys-R (t = 3.41, p<0.01). Among the latter three groups, no differences were observed (Figure 5.B). This pattern of results – the remediation groups(s) (Dys-R and Dys-RS for L.FFG-R.MOG iFC; Dys-RS for L.FFG-R.MPFC) being different from both TDC and the no remediation groups – is suggestive of compensatory cortical changes associated with remediation, rather than cortical normalization.

Examination of dimensional relationships between iFC and literacy competence further highlighted the contributions of these two L.FFG connections to literacy (scatter plots in Figure 5). First, iFC between L.FFG and R.MOG was positively correlated with literacy performance across dyslexia groups (Reading, R^2^ = 0.49, n = 33, p<0.001; Spelling R^2^ = 0.14, n = 33, p<0.05; Figure 5.A). When examining groups individually, iFC was positively correlated with reading scores in each remediation group (Dys-R, R^2^ = 0.55, n = 11, p<0.01; Dys-RS, R^2^ = 0.77, p<0.001), and with spelling scores within Dys-RS (R^2^ = 0.40, p<0.05). Such brain-behavior relationships were absent in the Dys-N and TDC groups. That is, children who exhibited stronger positive iFC between L.FFG and R.MOG tended to have higher scores in literacy performance across dyslexia groups, and this trend was particularly prominent in the remediation groups.

Second, iFC between L.FFG and R.MPFC was negatively correlated with literacy skills across dyslexia groups (Reading, R^2^ = 0.52, n = 33, p<0.001; Spelling, R^2^ = 0.33, n = 33, p<0.001; Figure 5.B). That is, children with dyslexia who exhibited more strongly negative iFC between L.FFG and R.MPFC tended to have higher literacy scores. When looking at groups individually, such brain-behavior relationships were present only within the full remediation group (Reading, R^2^ = 0.61, n = 11, p<0.01; Spelling, R^2^ = 0.50, n = 11, p<0.05), and absent in the TDC group and other dyslexia groups. This result with respect to the TDC group is consistent with our previous work, in which iFC between L.FFG and the medial prefrontal cortex negatively correlated with literacy performance in typical adults, but not in typically developing children [39].

**Figure 6:**
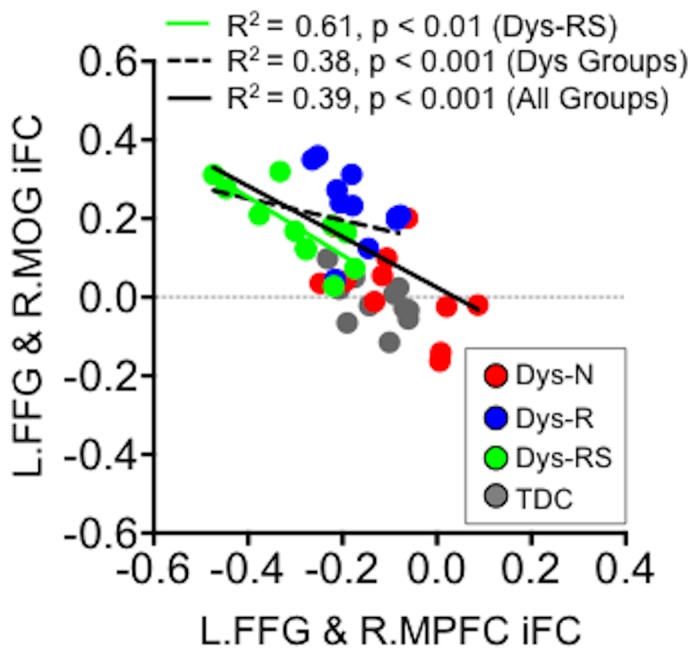
Relationship between the two putatively compensatory functional connections of the left fusiform gyrus (L.FFG) seed. The scatterplot represents the relationships between the two L.FFG connections (one with R.MOG and another with R.MPFC). iFC = intrinsic Functional Connectivity, R.MOG = Right Middle Occipital Gyrus, R.MPFC = Right Medial Prefrontal Cortex, Dys-N = Dyslexia with No Remediation, Dys-R = Dyslexia with Reading Remediation, Dys-RS = Dyslexia with Reading and Spelling Remediation, TDC = Typically Developing Children, Dys = Dyslexia: The correlation between these two L.FFG connections was significantly negative across all groups, across dyslexia groups, and in the full remediation group.

Given that L.FFG is involved in both functional connections (L.FFG-R.MOG, L.FFG-R.MPFC) associated with compensatory changes, an obvious question regards the relationship between these two sets of L.FFG connections. We examined their relationship (Figure 6). The correlation between L.FFG iFC with R.MOG and with R.MPFC was significantly negative across all groups (R^2^ = 0.39, n = 44, p<0.001) as well as across dyslexia groups (R^2^ = 0.38, n = 33, p<0.001). When examining groups individually, this relationship was significant in the full remediation group (Dys-RS, R^2^ = 0.61, n = 11, p<0.01). That is, children with stronger positive L.FFG-R.MOG connectivity were characterized by stronger negative iFC for the L.FFG-R.MPFC circuit, and this was particularly true in children in the full remediation group.

### Consideration of ADHD Symptomology as a Confound

To investigate potential effects of inattention and hyperactivity-impulsivity on the identified iFC of reading regions, we plotted relationships between iFC strength and scores of the inattentive and hyperactive-impulsive scores (CPRS DSM-IV), respectively. The iFC between L.IPS and L.MFG, within the dorsal attention network (e.g., [83]), was negatively correlated with inattention scores across all groups (R^2^ = 0.18, n = 44, p<0.01) (Figure S5.A.1). This brain-behavior relationship remained significant even after hyperactivity-impulsivity scores were covaried (R^2^ = 0.11, n = 44, p<0.05). Similarly, iFC between these regions was negatively correlated with hyperactivity-impulsivity scores across all groups (R^2^ = 0.26, n = 44, p<0.001) and across dyslexia groups (R^2^ = 0.20, n = 33, p<0.01) (Figure S5.A.2). These brain-behavior relationships remained significant after inattention scores were covaried (across all groups; R^2^ = 0.20, n = 44, p<0.01: across dyslexia groups; R^2^ = 0.18, n = 33, p<0.05). In examining brain-behavior correlations for L.IPS-L.MFG iFC in each group individually, only one significant correlation was observed: between L.IPS-L.MFG iFC and inattention scores in the Dys-R group, both before (R^2^ = 0.37, n = 11, p<0.05) (Figure S5.A.1) and after (R^2^ = 0.45, n = 11, p<0.05) covarying hyperactivity-impulsivity. For the two L FFG-based connections that differed among groups, neither exhibited a significant correlation with ADHD symptoms across all groups, although iFC between L.FFG and R.MOG was significantly correlated with inattention scores in the Dys-RS group before (R^2^ = 0.51, n = 11, p<0.05) (Figure S5.B.1) and after (R^2^ = 0.56, n = 11, p<0.05) covarying hyperactivity-impulsivity.

### Secondary Analysis

**Figure 7:**
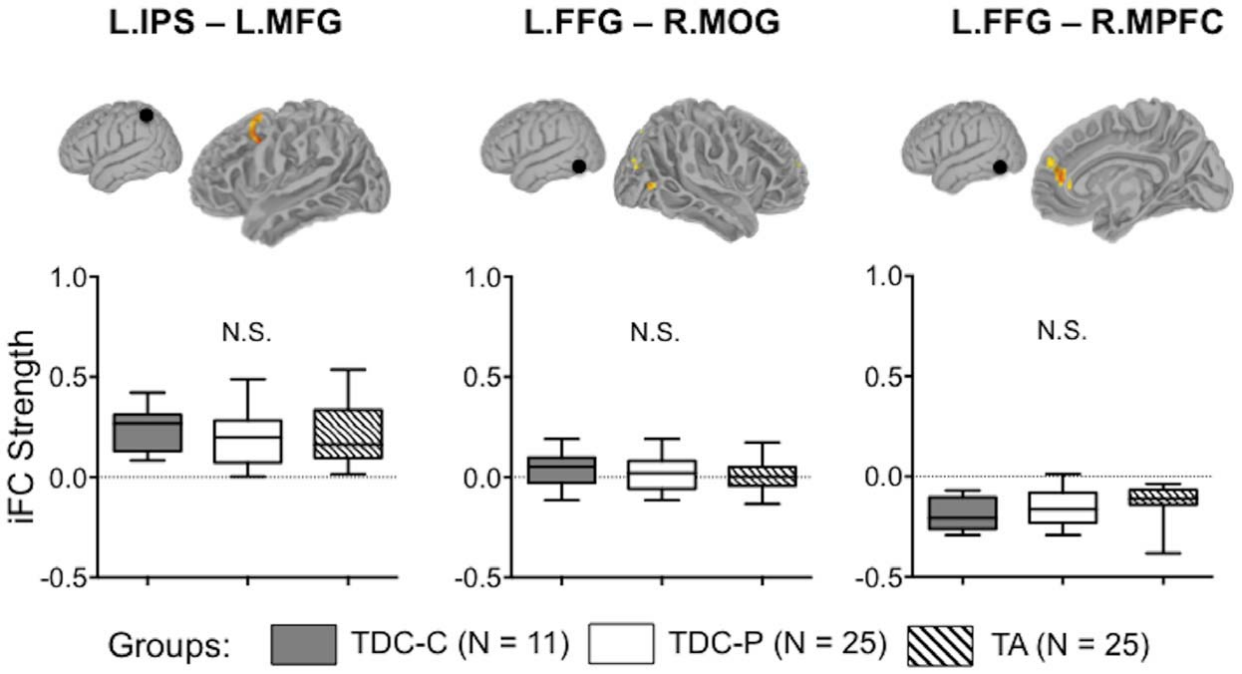
The strength of the identified iFC in two typically developing child groups and a typical adult group. iFC = Intrinsic Functional Connectivity, L.IPS = Left Intraparietal Sulcus, L.MFG = Left Middle Frontal Gyrus, L. FFG = Left Fusiform Gyrus, R.MOG = Right Middle Occipital Gyrus, R.MPFC = Right Medial Prefrontal Cortex, N.S. = Not Significant, TDC-C = Typically Developing Children in the Current work (n = 11), TDC-P = Typically Developing children in the Previous work (n = 25) [39], TA = Typical Adults (n = 25): Group differences were observed neither between the two TDC groups nor between TDC and TA groups.

A secondary analysis aimed to verify that the iFC patterns observed in the current TDC group are representative of normative/typical iFC profiles. As expected, pair-wise comparisons using unpaired t-tests revealed that, for all identified connections (L.IPS-L.MFG, L.FFG-R.MOG, and L.FFG-R.MPFC), there were no significant differences between the two TDC groups (Figure 7). This allows us to assert that the group differences observed in the present study reflect atypical/altered iFC patterns associated with dyslexia. In addition, for all identified connections, there were no significant differences between the TDC groups and the TA group (Figure 7), indicating that either they are not developmentally sensitive, or they are mature prior to age 7 (but see [39] for developmental differences for brain-behavior relationships with respect to L.FFG-R.MPFC iFC).

## Discussion

We identified group differences in the strength of intrinsic functional connectivity (iFC) of known reading regions among TDC and three dyslexia groups (i.e., no remediation, partial remediation, full remediation), with a particular focus on discerning potential effects of remediation. The findings are consistent with our first two predictions: 1) atypical iFC was associated with a history of dyslexia and 2) altered iFC consistent with cortical compensation associated with behavioral remediation. Specifically, the first finding suggests that dyslexia-related atypicality (i.e., reduced positive connectivity strength) in the L.IPS persists following remediation, even in fully remediated individuals. The second finding suggests that successful remediation of literacy deficits is associated with the emergence of compensatory changes in L.FFG iFC. Confidence in these results was bolstered by observing relationships between iFC strength and dimensional assessments of literary competence. However, we found no evidence supporting our third prediction, namely that we would find altered iFC reflecting cortical normalization. As discussed below, our findings, which complement those of task-based fMRI studies (e.g., [84]–[86]), should motivate the pursuit of longitudinal, multimodal (e.g., resting-state fMRI, task-based fMRI) studies with adequate sample size and systematic treatment of interventions.

### Atypical Connectivity Associated with Historical Diagnosis of Dyslexia

Our findings highlight the presence of dyslexia-related differences in iFC within the dorsal attention network [31], [83], [87]–[88]. Specifically, our analyses revealed reduced iFC between the left intraparietal sulcus (L.IPS) and left middle frontal gyrus (L.MFG) for all dyslexia groups, irrespective of remediation status. Notably, all children in the TDC group exhibited positive L.IPS-L.MFG connectivity, whereas the majority of the Dys-N group exhibited negative L.IPS-L.MFG connectivity (and thus this group had the weakest L.IPS-L.MFG iFC). Considering that brain regions involved in similar functions (i.e., co-activated by similar tasks) typically exhibit positive iFC [31], this result suggests that dysfunctional iFC between frontal and parietal regions within the attention network characterizes a history of dyslexia.

Consistent with its hypothesized role in attentional function, weaker iFC in this circuit was also associated with higher parental ratings of inattention and of hyperactivity-impulsivity, both of which were negatively correlated with children’s reading competence (i.e., the more elevated the ADHD-related symptoms, the poorer the reading performance). Persistence of weaker L.IPS-L.MFG iFC, even among highly remediated individuals, suggests that interventions do not directly address alterations within the dorsal attention network. This is not surprising, as current interventions tend to exclusively focus on phonetics and word processing and ignore attentional function. Our findings support recent calls for an increased focus on attention as a potential target for intervention [81], [89]–[90].

### Cortical Compensation Associated with Behavioral Remediation

A key question in the dyslexia literature is the extent to which intervention efforts “cure” the underlying deficit or invoke alternative, compensatory processing strategies capable of overcoming innate deficiencies [23], [91]–[94]. Our findings suggest the latter with respect to the iFC between the left fusiform gyrus (L.FFG) and regions in the right hemisphere outside the known reading network. L.FFG, part of the ventral visual pathway and known as the Visual Word Form Area [95]–[96], exhibits reduced activity (e.g., [86], [97]–[98]) and reduced functional connectivity with other language regions [85], [99]–[100] in individuals with dyslexia. Our findings indicate that L.FFG may exhibit compensatory changes associated with behavioral remediation (i.e., normalized literacy skills). Specifically, we observed two patterns of change that may reflect these compensatory effects. First, iFC between the L.FFG and the right middle occipital gyrus (R.MOG), a component of the dorsal visual pathway [101]–[102], was increased in both remediation groups. Second, the full remediation group (Dys-RS) exhibited stronger negative iFC between the L.FFG and the right medial prefrontal cortex (R.MPFC), a core region of the default network [103]–[105]. This pattern – the remediation groups (i.e., only in the Dys-RS group for L.FFG-R.MPFC iFC) being different from the TDC group and Dys-N group – is more likely to reflect cortical compensation than normalization. Of note, the strength of these two functional connections based in the L.FFG was strongly correlated within the full remediation group, further supporting the suggestion that L.FFG serves as a hub region for compensatory changes in the dyslexic brain.

Importantly, the two observed connections of L.FFG are relevant to literacy competence. First, stronger coupling between posterior visual regions (L.FFG-R.MOG) was associated with better literacy performance across dyslexic children, particularly those in the remediation groups. This atypically increased functional coupling within the visual network may suggest greater reliance on visual reading strategies to compensate for weak phonological processing skills (i.e., phonological weakness was documented in prior psychoeducational evaluations for children in the two remediation groups, although the majority of them showed no phonological deficits in the current assessment). Our interpretation is supported by reading errors made by children in the remediation groups, such as misreading of visually similar words (e.g., “useless” pronounced as “unless”). Of note, reading errors for visually similar words are reported to be common in dyslexic individuals [79], even in those who exhibit good reading comprehension [106].

The second observation with respect to L.FFG’s behavioral relevance is that stronger negative connectivity or functional segregation between task-positive and task-negative default networks/regions (L.FFG and R.MPFC) was associated with better literacy performance across dyslexic children, particularly those in the full remediation group. This brain-behavior relationship – the more negative the iFC, the better the literacy performance – is characteristic of typical adult readers [39]. Given that functional segregation from the default network is thought to support efficient cognitive processing in typical adults [107], stronger negative iFC between L.FFG-R.MPFC and its adult-like relationships with literacy competence in the full remediation group may reflect increased behavioral efficiency, as a compensatory mechanism, following effective remediation. This finding suggests the merits of expanding models of dyslexia to include interactions between task-positive or reading networks and the default network [39]. Although negative iFC has been reported by a number of studies (e.g., [108]), its underlying physiological mechanisms remain a topic of research. Thus, longitudinal studies are required to test our interpretations and to further clarify the roles of negative iFC/functional segregation between task-active and task-negative default regions/networks in clinical populations, particularly with respect to remediation effects.

### Limitations

The results of this study must be interpreted in light of its limitations. First, we employed an observational, cross-sectional design, rather than a longitudinal approach. This raises the question of whether there were differences in literacy performance between the dyslexia groups that preceded their intervention training, potentially explaining the patterns observed in the present study. Although we cannot rule out the presence of subtle pre-existing differences between the groups, the level of literacy performance prior to any intervention in children of the remediation groups was grossly similar to that of the current literacy performance in children of the no remediation group. That is, we obtained evidence that children in both remediation groups had standard scores lower than 85 on a standardized literacy test at the time of their prior assessment and diagnosis; children in the no remediation group (i.e., no experience of intervention training) currently had standard scores lower than 85 on both the WIAT Word Reading and the WIAT Spelling subscales when assessed as part of this study. Nevertheless, cortical changes associated with remediation would best be captured by longitudinal studies involving coordinated interventions for dyslexia, as remediation is not instantaneous, but instead occurs over time. Still, our findings should motivate continued examination of cortical signatures of different dyslexia profiles, assessed by multiple approaches, including R-fMRI, before, during, and after remediation efforts. The second limitation derives from the modest size of the individual dyslexia groups. Despite the modest sample size, we were able to stratify individuals with dyslexia on the basis of remediation status. This enhanced our statistical power to detect group differences [109]. Third, this study lacked one type of dyslexia group: children with a history of dyslexia diagnosis, who completed intervention training but remained deficient in reading skills [110]. Although a few children screened in the current study exhibited literacy deficits after having intervention training (confirmed by a previous clinical documentation and/or informal interview with parents), they were excluded due to their low IQ scores, which typically preclude diagnosis of dyslexia. It is worth noting that this widely applied diagnostic criterion based on the IQ-reading discrepancy has been criticized and remains a topic of investigation [111]–[112]. Future research is urgently needed to elucidate potential differences in reading-related iFC patterns between responders and non-responders (including those with low IQ) to intervention training. Finally, the present investigation was limited to R-fMRI data. Although this approach has been fruitful, the combination of task-based and task-independent modalities holds the greatest potential for the further characterization and interpretation of deficits, compensatory responses, and functional normalization in dyslexia.

### Conclusions

Using task-independent R-fMRI, we delineated candidate cortical signatures of distinct dyslexia profiles. Reduced intrinsic functional connectivity within the dorsal attention network is associated with a history of dyslexia, calling for further focus on the role of attention in dyslexia and on targeted interventions, whereas the left fusiform gyrus in the ventral visual pathway appears to be a hub for compensatory mechanisms in dyslexia remediation. Intrinsic functional connectivity approaches appear to provide a firm basis for investigating the cortical correlates of dyslexia, for monitoring dynamic changes associated with behavioral remediation in individuals with dyslexia, and eventually for evaluating the effectiveness of dyslexia remediation.

## Supporting Information

Figure S1 **Mean Framewise Displacement (FD).** The box-and-whisker plot (A) depicts mean FC for each group and no group differences, whereas the scatter plot (B) depicts no relationship between age and mean FD. Dys-N = Dyslexia with No Remediation, Dys-R = Dyslexia with Reading Remediation, Dys-RS = Dyslexia with Reading and Spelling Remediation, TDC = Typically Developing Children.
(TIF)

Figure S2 **Conners’ Parent Rating Scales of DSM-IV Symptoms of Inattention and Hyperactivity-Impulsivity.** The box-and-whisker plots depict (A.1) inattention and (A.2) hyperactivity-impulsivity scores for each group and group differences. The scatterplots represent relationships of WIAT Word Reading scores with (B.1) inattention and (B.2.) hyperactivity-impulsivity scores. Dys-N = Dyslexia with No Remediation, Dys-R = Dyslexia with Reading Remediation, Dys-RS = Dyslexia with Reading and Spelling Remediation, TDC = Typically Developing Children, ss = standard scores, **p<0.01, *p<0.05: The mean scores in Inattention and Hyperactivity-Impulsivity subscales were significantly lower in TDC than the dyslexia groups, and significant negative correlations between attention (i.e., both inattention and hyperactivity-impulsivity) and reading scores were observed across all groups.
(TIF)

Figure S3 **Effects of five different statistical models with respects to global signal regression (GSreg), framewise displacement regression (FDreg), and data scrubbing (Scrub).** +GSreg = analyzed with GSreg, -GSreg = analyzed without GSreg, +FDreg = analyzed with FDreg, -FDreg = analyzed without FDreg, +Scrub = frames larger than 0.5FD were Scrubbed from the dataset, iFC = Intrinsic Functional Connectivity, L.IPS = Left Intraparietal Sulcus, L.FFG = Left Fusiform Gyrus, R.MOG = Right Middle Occipital Gyrus, R.MPFC = Right Medial Prefrontal Cortex, Dys-N = Dyslexia with No Remediation, Dys-R = Dyslexia with Reading Remediation, Dys-RS = Dyslexia with Reading and Spelling Remediation, TDC = Typically Developing Children: The results from the primary analysis (+GSreg+FDreg) were replicated in other analyses, although those yielded from the model where the global signal was not regressed (i.e., -GSreg+FDreg and -GSreg-FDreg) were significant only at uncorrected p<0.05.
(TIF)

Figure S4 **Region-specific effects of global signal regression on the group difference in intrinsic functional connectivity.** L.IPS = left intraparietal suclus, L.FFG = left fusiform gyrus, R. MOG = right middle occipital gyrus, R.MPFC = right medial prefrontal cortex**,** +GSreg+FDreg = analyzed with both GSreg and FDreg, -GSreg+FDreg = analyzed without GSreg but with FDreg: Z >2.3, cluster significance, p<0.05, corrected; With no global signal regression, the L.FFG seed failed to show the group differences identified in the primary analysis where global signal was regressed (A: R.MOG and B: R.MPFC).
(TIF)

Figure S5 **Relationships between each iFC and ADHD-symptoms (**Conners’ Parent Rating Scales; DSM-IV Symptoms of Inattention and Hyperactivity-Impulsivity). The upper scatterplots depict relationships between iFC and inattention scores (A.1, B.1, and C.1), whereas the lower ones depict relationships between iFC and hyperactivity-impulsivity scores (A.2, B.2, and C.2). iFC = intrinsic Functional Connectivity, L.IPS = Left Intraparietal Sulcus, L.FFG = Left Fusiform Gyrus, R.MOG = Right Middle Occipital Gyrus, R.MPFC = Right Medial Prefrontal Cortex, Dys-N = Dyslexia with No Remediation, Dys-R = Dyslexia with Reading Remediation, Dys-RS = Dyslexia with Reading and Spelling Remediation, TDC = Typically Developing Children, Dys = Dyslexia: The iFC between L.IPS and L.MFG was negatively correlated with inattention scores as well as hyperactivity-impulsivity scores across all groups.
(TIF)

Materials and Methods S1 **Dyslexia sample characterization.**
(DOCX)
