# Supplementary figures and images for: Cortical Signatures of Dyslexia and Remediation: An Intrinsic Functional Connectivity Approach
Source: PLoS One. 2013 Feb 11;8(2):e55454. doi: 10.1371/journal.pone.0055454 (PMC3569450; doi:10.1371/journal.pone.0055454)

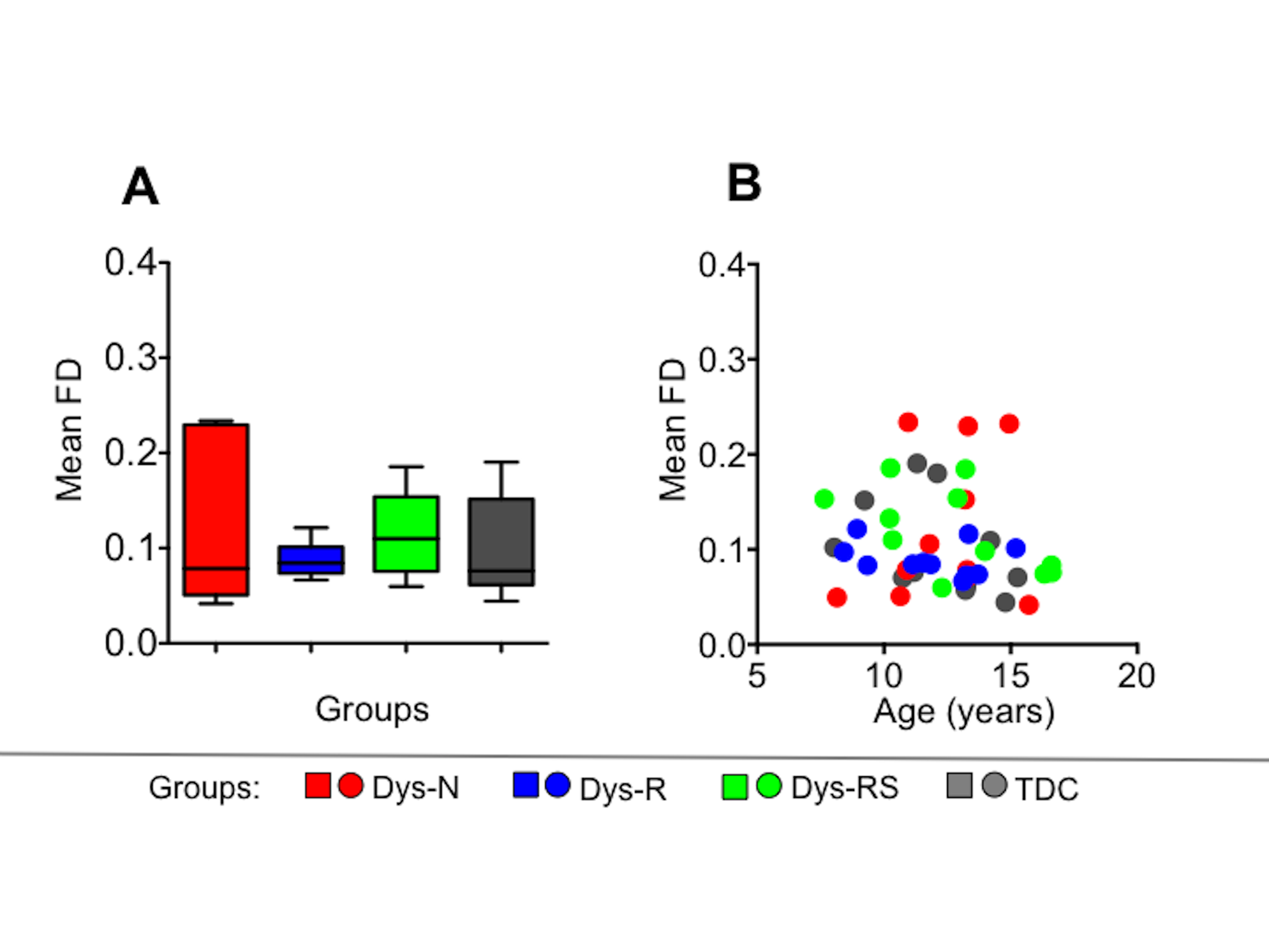

Supplement: Figure S1 — Mean Framewise Displacement (FD). The box-and-whisker plot (A) depicts mean FC for each group and no group differences, whereas the scatter plot (B) depicts no relationship between age and mean FD. Dys-N = Dyslexia with No Remediation, Dys-R = Dyslexia with Reading Remediation, Dys-RS = Dyslexia with Reading and Spelling Remediation, TDC = Typically Developing Children. (TIF) [file pone.0055454.s001.tif]

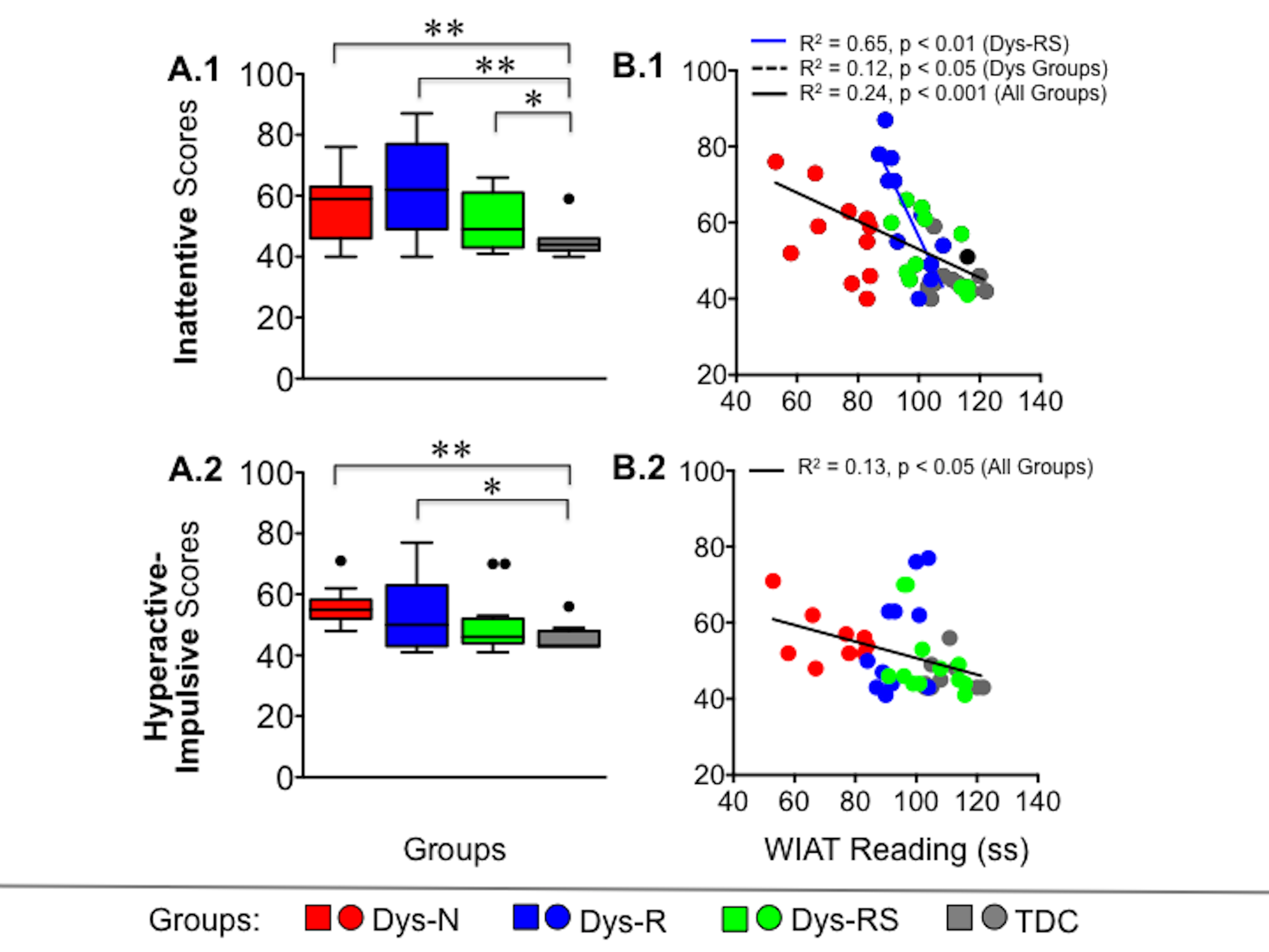

Supplement: Figure S2 — Conners’ Parent Rating Scales of DSM-IV Symptoms of Inattention and Hyperactivity-Impulsivity. The box-and-whisker plots depict (A.1) inattention and (A.2) hyperactivity-impulsivity scores for each group and group differences. The scatterplots represent relationships of WIAT Word Reading scores with (B.1) inattention and (B.2.) hyperactivity-impulsivity scores. Dys-N = Dyslexia with No Remediation, Dys-R = Dyslexia with Reading Remediation, Dys-RS = Dyslexia with Reading and Spelling Remediation, TDC = Typically Developing Children, ss = standard scores, **p<0.01, *p<0.05: The mean scores in Inattention and Hyperactivity-Impulsivity subscales were significantly lower in TDC than the dyslexia groups, and significant negative correlations between attention (i.e., both inattention and hyperactivity-impulsivity) and reading scores were observed across all groups. (TIF) [file pone.0055454.s002.tif]

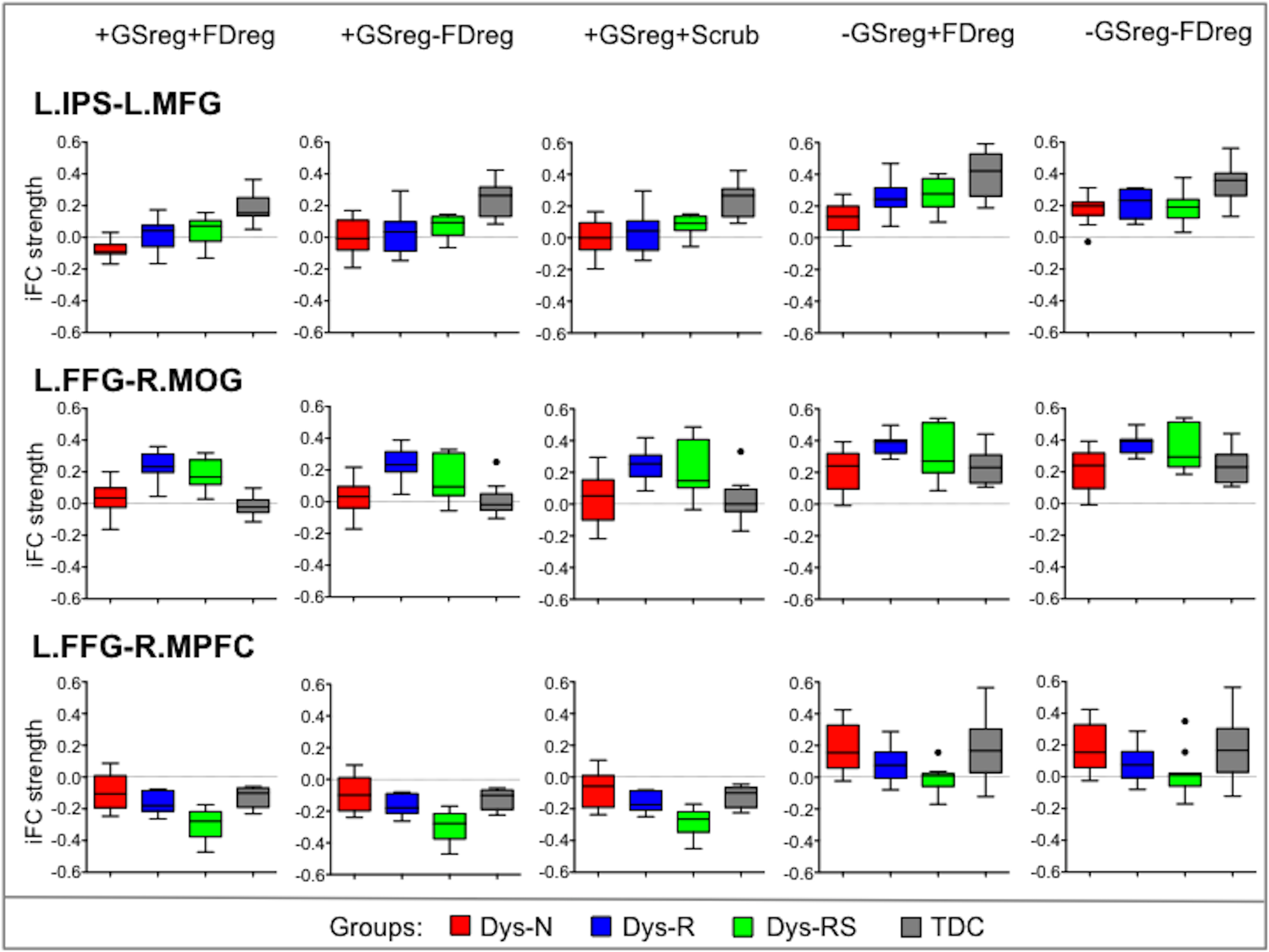

Supplement: Figure S3 — Effects of five different statistical models with respects to global signal regression (GSreg), framewise displacement regression (FDreg), and data scrubbing (Scrub). +GSreg = analyzed with GSreg, -GSreg = analyzed without GSreg, +FDreg = analyzed with FDreg, -FDreg = analyzed without FDreg, +Scrub = frames larger than 0.5FD were Scrubbed from the dataset, iFC = Intrinsic Functional Connectivity, L.IPS = Left Intraparietal Sulcus, L.FFG = Left Fusiform Gyrus, R.MOG = Right Middle Occipital Gyrus, R.MPFC = Right Medial Prefrontal Cortex, Dys-N = Dyslexia with No Remediation, Dys-R = Dyslexia with Reading Remediation, Dys-RS = Dyslexia with Reading and Spelling Remediation, TDC = Typically Developing Children: The results from the primary analysis (+GSreg+FDreg) were replicated in other analyses, although those yielded from the model where the global signal was not regressed (i.e., -GSreg+FDreg and -GSreg-FDreg) were significant only at uncorrected p<0.05. (TIF) [file pone.0055454.s003.tif]

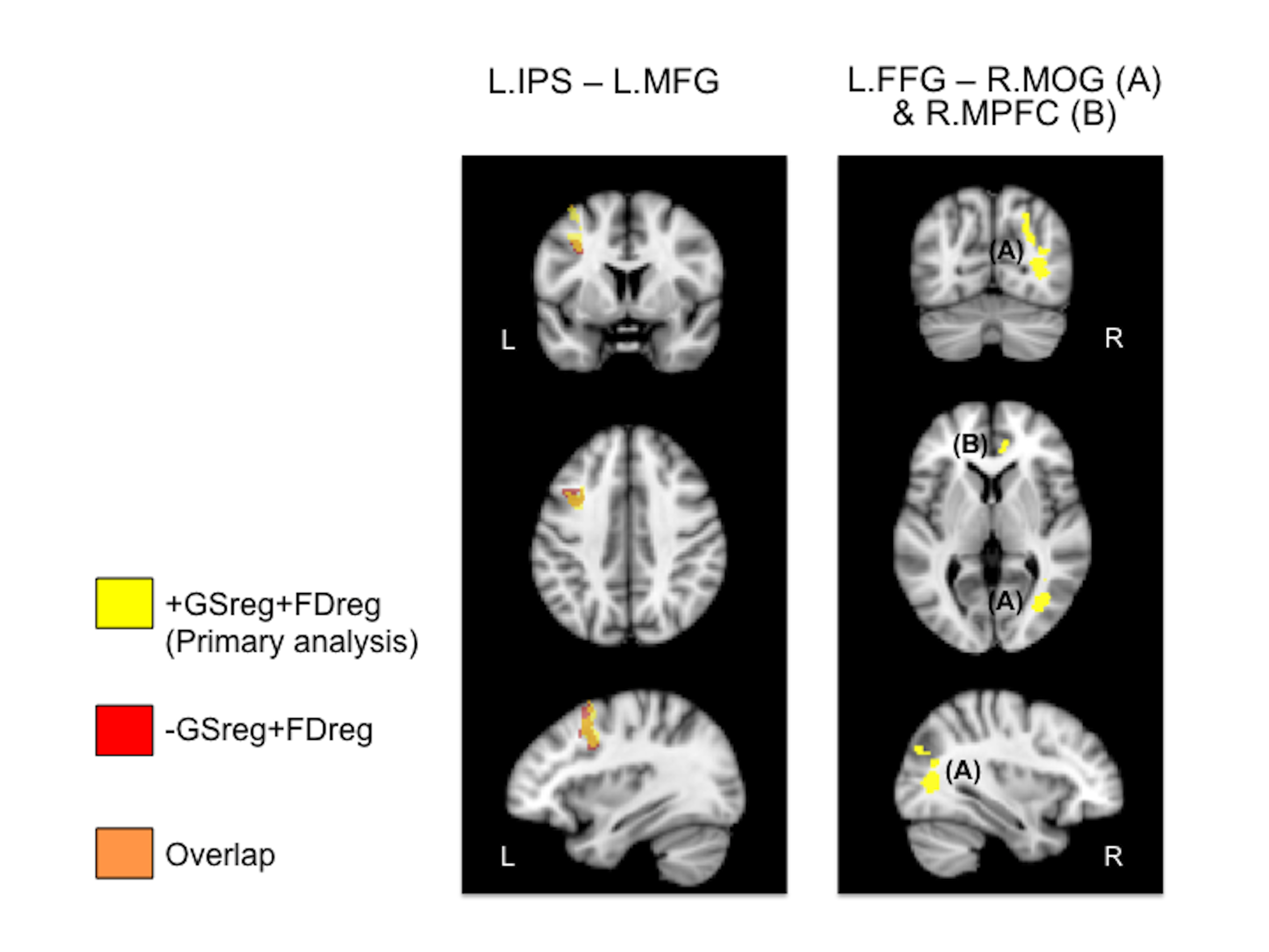

Supplement: Figure S4 — Region-specific effects of global signal regression on the group difference in intrinsic functional connectivity. L.IPS = left intraparietal suclus, L.FFG = left fusiform gyrus, R. MOG = right middle occipital gyrus, R.MPFC = right medial prefrontal cortex, +GSreg+FDreg = analyzed with both GSreg and FDreg, -GSreg+FDreg = analyzed without GSreg but with FDreg: Z >2.3, cluster significance, p<0.05, corrected; With no global signal regression, the L.FFG seed failed to show the group differences identified in the primary analysis where global signal was regressed (A: R.MOG and B: R.MPFC). (TIF) [file pone.0055454.s004.tif]

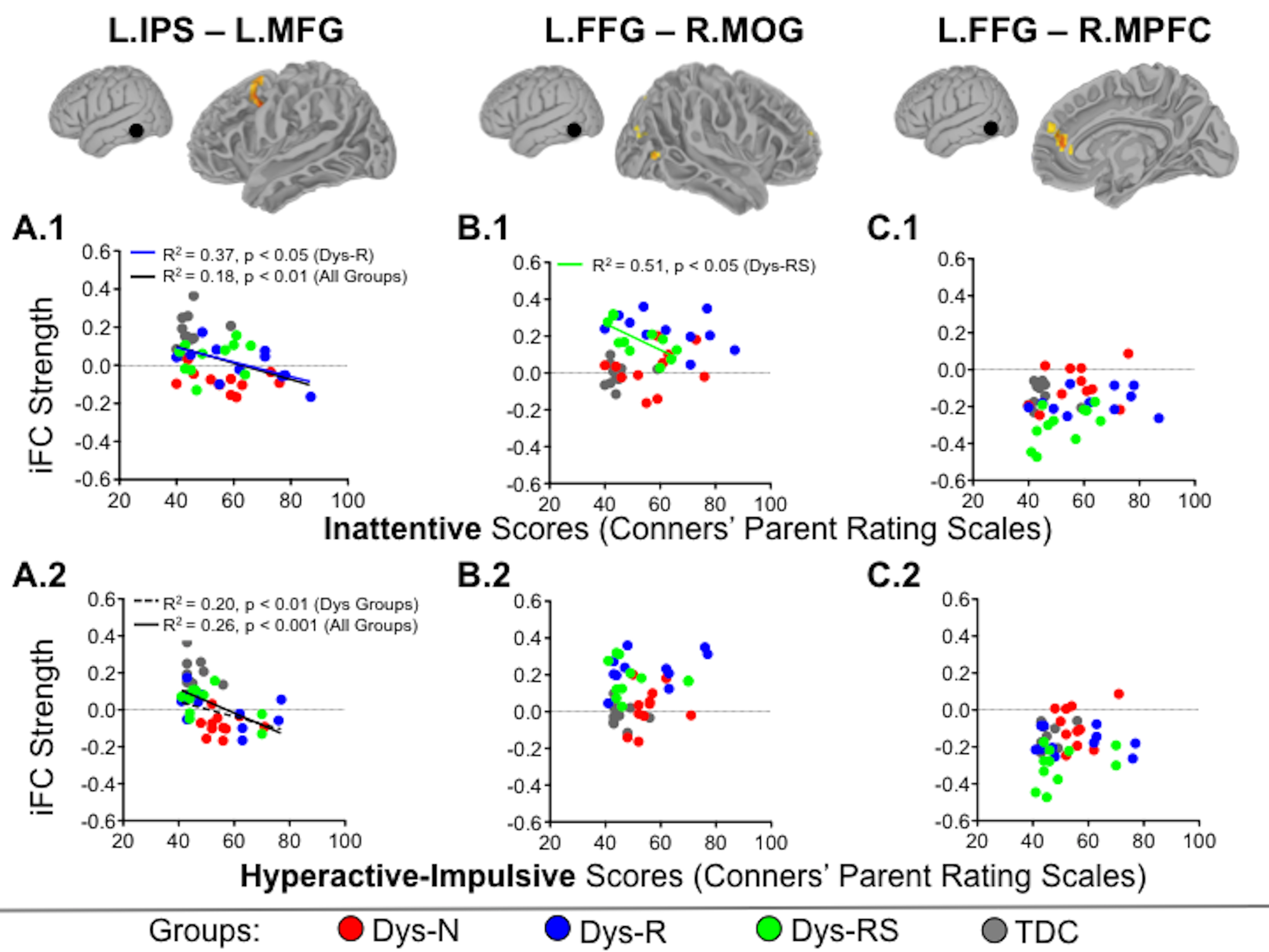

Supplement: Figure S5 — Relationships between each iFC and ADHD-symptoms (Conners’ Parent Rating Scales; DSM-IV Symptoms of Inattention and Hyperactivity-Impulsivity). The upper scatterplots depict relationships between iFC and inattention scores (A.1, B.1, and C.1), whereas the lower ones depict relationships between iFC and hyperactivity-impulsivity scores (A.2, B.2, and C.2). iFC = intrinsic Functional Connectivity, L.IPS = Left Intraparietal Sulcus, L.FFG = Left Fusiform Gyrus, R.MOG = Right Middle Occipital Gyrus, R.MPFC = Right Medial Prefrontal Cortex, Dys-N = Dyslexia with No Remediation, Dys-R = Dyslexia with Reading Remediation, Dys-RS = Dyslexia with Reading and Spelling Remediation, TDC = Typically Developing Children, Dys = Dyslexia: The iFC between L.IPS and L.MFG was negatively correlated with inattention scores as well as hyperactivity-impulsivity scores across all groups. (TIF) [file pone.0055454.s005.tif]
