# Supplementary material for: Cortical Signatures of Dyslexia and Remediation: An Intrinsic Functional Connectivity Approach
Source: PLoS One. 2013 Feb 11;8(2):e55454. doi: 10.1371/journal.pone.0055454 (PMC3569450; doi:10.1371/journal.pone.0055454)
Supplement: Materials and Methods S1 — Dyslexia sample characterization. (DOCX) [file pone.0055454.s006.docx]

**Supporting Materials and Methods**

**Materials and Methods S1. Dyslexia sample characterization**

***Literacy measures for all groups***

One-way ANOVA showed that the four groups (i.e., TDC, Dys-N, Dys-R, and Dys-RS) differed significantly on literacy measures, as expected (Table 1 and Figure 2): WIAT Word Reading, F _(3, 40)_ = 34.06, p < 0.001; WIAT Spelling, F _(3, 40)_ = 61.33, p < 0.001; TOWRE SWE, F _(3, 40)_ = 10.00, p < 0.001; TOWRE PDE, F _(3, 40)_ = 15.81, p < 0.001. Post-hoc analysis using unpaired t tests revealed that the Dys-N group exhibited significantly poorer performance on the majority of the literacy measures relative to TDC (WIAT Word Reading, t = 8.96, p < 0.001; WIAT Spelling, t = 10.68, p < 0.001; TOWRE SWE, t = 4.81, p < 0.001; TOWRE PDE, t = 5.86, p < 0.001) and relative to the two remediation groups (Relative to Dys-R: WIAT Word Reading, t = 5.45, p < 0.001; WIAT Spelling, t = 1.20, not significant; TOWRE SWE, t = 3.23, p < 0.01; TOWRE PDE, t = 3.11, p < 0.01, Relative to Dys-RS: WIAT Word Reading, t = 6.71, p < 0.001; WIAT Spelling, t = 4.80, p < 0.001; TOWRE SWE, t = 3.69, p < 0.01; TOWRE PDE, t = 3.31, p < 0.01). With regard to spelling competence (WIAT Spelling), all dyslexia groups exhibited significantly poorer performance than the TDC group (Dys-N: t = 10.68, p < 0.001; Dys-R: t = 13.51, p < 0.001; Dys-RS: t = 6.46, p < 0.001).

Although children in the partial remediation group (Dys-R) exhibited normal (i.e., standard scores higher than 85) reading accuracy and decoding, their scores remained significantly lower than those of the TDC group (WIAT Word Reading, t = 4.54, p < 0.001; TOWRE PDE, t = 4.28, p < 0.001). Similarly, despite falling within the normal range for spelling, the performance of children in the full remediation group (Dys-RS) remained significantly lower than that of the TDC (WIAT Spelling, t = 6.46, p < 0.001).

***Literacy measures only for the dyslexia groups***

On the WIAT Reading Comprehension, the performance of all three dyslexia groups fell within the normal range (i.e., within 1.5 standard deviation of the mean). On CTOPP measures, the full remediation group exhibited no deficit but the other dyslexia groups exhibited mild deficits in these phonological measures; In the Dys-R group, the performance of CTOPP Nonword Repetition fell within the lower average range, whereas the performance of both CTOPP Elision and CTOPP Nonword Repetition fell within the lower average range in the Dys-N group.

Significant group differences were noted on the WIAT Reading Comprehension, (F _(2, 30)_ = 9.37, p < 0.001), on CTOPP Elision (F _(2, 30)_ = 13. 46, p < 0.001), and CTOPP Nonword Repetition (F _(2, 30)_ = 3.64, p < 0.05). Post-hoc analysis using unpaired t-tests revealed that the Dys-N group exhibited significantly poorer performance on these measures than other dyslexia groups. Post-hoc analysis revealed that on the WIAT Reading Comprehension, the Dys-N and Dys-R groups exhibited significantly poorer performance than Dys-RS group (t = 3.63, p < 0.01 and t = 3.25, p < 0.01, respectively). On CTOPP Elision, the Dys-N group exhibited significantly poorer performance than Dys-R (t = 5.87, p < 0.001) and Dys-RS (t = 3.81, p < 0.01) groups. On CTOPP Nonword Repetition, a group difference was observed only between the Dys-N and Dys-RS groups (t = 2.69, p < 0.05).

***Post-hoc analysis on ADHD symptoms***

One-way ANOVA showed a significant main effect of group for both inattention (F _(3, 40)_ = 5.30, p < 0.01) and hyperactivity-impulsivity (F _(3, 40)_ = 3.02, p < 0.05) scores. Post-hoc analysis using an unpaired t-test revealed that the mean inattention score was significantly lower in TDC (44.8 ± 5.1) than the dyslexia groups; Dys-N (57.1 ± 11.3), t = 3.28, p < 0.01; Dys-R (62.6 ± 15.2), t = 3.68, p < 0.01; Dys-RS (53.4 ± 9.4), t = 2.35 p < 0.05 (Supplementary Figure 2.A.1). Similarly, the mean hyperactivity-impulsivity score was significantly lower in TDC (45.5 ± 4.1) relative to Dys-N (56.0 ± 6.5), t = 2.9, p < 0.05 and Dys-R (55.4 ± 13.4), t = 3.5, p < 0.01, but no significant difference was observed between TDC and Dys-RS (50.8 ± 9.9), t = 1.67 (Supplementary Figure 2.A.2). Among the dyslexia groups, no significant group difference was noted for either inattention (F _(2, 30)_ = 1.95, not significant) or hyperactivity-impulsivity (F _(2, 30)_ = 0.79, not significant) scores.
